# Comparative Study on Modulation of Synchronized Neuronal Activity by SK Channels and Na/K-ATPase

**DOI:** 10.3390/ijms262010004

**Published:** 2025-10-14

**Authors:** Dmitry A. Sibarov, Sergei I. Boikov, Tatiana V. Karelina, Vadim V. Yushko, Alyona I. Fedorina, Sergei M. Antonov

**Affiliations:** Sechenov Institute of Evolutionary Physiology and Biochemistry, Russian Academy of Sciences, Torez pr. 44, Saint-Petersburg 194223, Russia

**Keywords:** epilepsy, epileptiform activity, SK channels, CyPPA, Na/K-ATPase, ouabain, neurons, calcium

## Abstract

Drug-resistant epilepsy remains a therapeutic challenge, requiring new molecular targets beyond conventional antiepileptic drugs. Small-conductance calcium-activated potassium (SK) channels and Na/K-ATPase (NKA) contribute to afterhyperpolarization via distinct mechanisms, offering complementary ways to suppress hyperexcitability. We examined SK activation and NKA modulation in synchronized epileptiform activity in a primary culture of cortical neurons obtained from rat embryos. Epileptiform discharges were induced by magnesium-free solution and assessed by patch-clamp and calcium imaging. The SK2/3 activator CyPPA (10 µM) reduced epileptiform current (EC) amplitude and integral and decreased synchronized calcium transient (CT) frequency but gradually elevated basal calcium. In contrast, ouabain (1 nM), a selective modulator of high-affinity NKA isoforms, attenuated EC amplitude, strongly suppressed CTs, and showed persistent effects after washout, accompanied by asynchronous glial calcium activity. Co-application of CyPPA with ouabain abolished CyPPA-induced calcium elevation while maintaining suppression of neuronal synchrony. The broader SK/IK activator NS309 (10 µM) reduced CT frequency and basal calcium without affecting glia. Thus, SK activation and NKA signaling suppress epileptiform synchronization through distinct yet convergent pathways: SK channels via afterhyperpolarization and NKA via afterhyperpolarization and calcium-dependent signaling. Their combination enhances efficacy and prevents adverse calcium buildup, supporting SK–NKA co-targeting as a strategy against drug-resistant epilepsy.

## 1. Introduction

Epilepsy is one of the most common neurological disorders. Despite the availability of pharmacological treatments, approximately 30% of individuals with epilepsy exhibit resistance to conventional antiepileptic drugs (AEDs) [1,2]. Therefore, the development of AEDs with novel mechanisms of action remains a key therapeutic priority for managing drug-resistant epilepsy.

Current pharmacological treatments for epilepsy primarily target neuronal excitability and include several mechanistic classes: sodium channel blockers (Phenytoin, Carbamazepine, Oxcarbazepine, Lamotrigine, Lacosamide); calcium channel blockers (Ethosuximide, Gabapentin, Pregabalin); GABAergic enhancers (Benzodiazepines, Phenobarbital, Tiagabine, Vigabatrin); glutamate receptor antagonists (Perampanel, Topiramate); synaptic vesicle modulators (Levetiracetam, Brivaracetam); KCNQ/Kv7 potassium channel openers (Retigabine) [3,4,5].

Retigabine, which enhances potassium channel activity to promote neuronal hyperpolarization, was withdrawn from the market due to adverse side effects. Topiramate was shown to hyperpolarize neurons [6], but it targets multiple ion channels and enzymes, making it hard to evaluate the contribution of a specific mechanism to its antiepileptic effects.

However, hyperpolarization remains a promising strategy to reduce neuronal hyperexcitability, and additional molecular targets in this pathway are now under investigation. Two emerging targets are small-conductance calcium-activated potassium (SK) channels and Na/K-ATPase (NKA). Both contribute to afterhyperpolarization (AHP) in neurons though through distinct mechanisms.

SK channels are activated by intracellular Ca^2+^ influx during action potentials. They mediate medium-duration AHP (mAHP), regulating spike frequency adaptation and neuronal excitability [7,8].

Na/K-ATPase (NKA) restores ionic gradients after neuronal firing. Its electrogenic activity contributes to slow AHP (sAHP) and maintains longer-term excitability homeostasis [9,10].

In this study, we investigate a dual-mechanism strategy to suppress synchronized epileptiform activity in primary cortical neuronal cultures. We utilize two agents that enhance distinct hyperpolarizing and calcium-signaling pathways.

CyPPA, a subtype-selective positive modulator of SK2 and SK3 channels, shifts the Ca^2+^ activation curve to lower thresholds, allowing SK channels to open even at resting intracellular Ca^2+^ concentrations [11]. This activation induces fast afterhyperpolarizing currents that truncate action potentials and suppress burst firing—an established mechanism for dampening synchronized neuronal bursts. A concentration of 10 µM CyPPA or NS309 in our study lies within an appropriate range for the selective activation of neuronal SK channels. At this concentration, both modulators consistently potentiate SK2 and SK3 channels—the predominant subtypes in neurons—while SK1 and IK (KCa3.1) channels remain largely unaffected due to their minimal neuronal expression. Notably, the EC_50_ (14 and 5.6 µM for SK2 and SK3, respectively) for CyPPA was determined at an intracellular Ca^2+^ concentration of 200 nM [11]. During synchronized neuronal activity, intracellular Ca^2+^ levels rise, which shifts the apparent EC_50_ to lower values. Consequently, during epileptiform activity, even lower CyPPA concentrations can effectively activate SK channels. Therefore, 10 µM is sufficient to achieve robust SK2/3 potentiation in neurons while avoiding the excessive Ca^2+^ elevations and off-target effects associated with higher doses [12].

Ouabain at low-nanomolar concentrations selectively targets high-affinity α2/α3 isoforms of NKA and triggers multiple intracellular signaling pathways [13]. Positive modulation of NKA activity by 0.1–1 nM ouabain [14] may potentially enhance slow, electrogenic hyperpolarization and shift ionic homeostasis. In hippocampal neuron–astrocyte co-cultures, 1–10 nM ouabain has been shown to modulate intracellular Ca^2+^ dynamics [14,15,16,17,18] and stimulate the sodium–calcium exchanger [14], ultimately reducing neuronal excitability [9,18]. Although ouabain is rarely used clinically due to poor pharmacokinetics [19] and a narrow therapeutic index, it is the most extensively studied cardiotonic steroid. The 1 nM ouabain concentration used in our study is well below therapeutic levels, aligns with physiological levels of endogenous cardiotonic steroids [20], and has been shown to positively modulate NKA activity [13] and to activate multiple NKA-mediated signaling pathways [16,21].

Early-stage AED screening traditionally relied on in vivo models, but in vitro systems now provide greater efficiency and experimental control. Dissociated neuronal cultures and organotypic hippocampal slices are widely used to study epileptiform responses, often combined with electrophysiology and calcium imaging to probe AED mechanisms on ion channels and receptors. Primary cortical or hippocampal cultures are particularly valuable, as they generate synchronized miniature excitatory postsynaptic currents (mEPSCs), reflecting mature synaptic connectivity and network organization [22]. These cultures also display synchronized calcium oscillations driven by spontaneous action potential bursts, a hallmark of self-organized neuronal activity [23,24]. Such oscillations provide a robust platform for the phenotypic screening of candidate AEDs [25,26].

One of the commonly used in vitro models of epileptiform activity involves incubating cultures in magnesium-free (Mg^2+^-free) extracellular solution, which enhances NMDA receptor activity and mimics status epilepticus-like neuronal hyperactivity [27,28].

In our work, we induced synchronized epileptiform activity in primary cortical neurons using a magnesium-free solution. To evaluate the antiepileptiform efficacy of CyPPA and ouabain, we employed two complementary experimental approaches: patch-clamp electrophysiology, to quantify changes in the frequency and amplitude of epileptiform postsynaptic currents in individual neurons, and calcium imaging, to visualize and analyze network-level synchronous activity at both the single-cell and population scales, revealing how hyperpolarizing agents affect ensemble dynamics. Together, these methods allowed us to assess how modulation of two distinct hyperpolarization mechanisms impacts synchronized neuronal activity, offering insight into potential combinatory strategies for treating drug-resistant epilepsy.

## 2. Results

### 2.1. NKA Signaling and SK-Channel Activation Suppress Synchronized Epileptiform Currents

**Figure 1:**
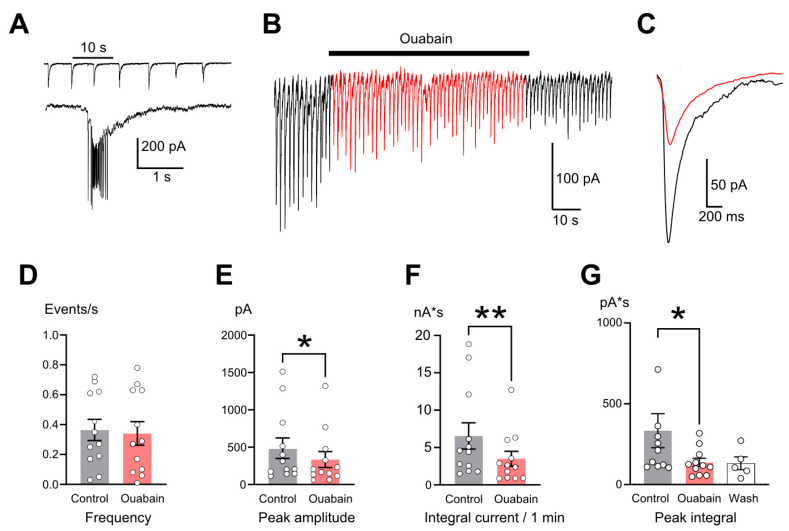
Ouabain reduces the magnitude of epileptiform currents (ECs). (**A**) Typical recording of periodic postsynaptic ECs. Expanded view shows spike events generated by action potentials in a presynaptic neuron on the top of a single EC. (**B**) A recording illustrating the developments of the effect of ouabain (1 nM) on ECs. Application of ouabain is shown as a bar above the trace. (**C**) An overlap of EPSCs averaged from intervals before (black) and after 1 min of ouabain application (red). (**D**–**G**) Comparison of mean ± SEM values of EC frequency (**D**), EC peak amplitudes (**E**), integral current per 1 min (**F**), and mean individual EC integral (**G**) before (gray, control), during application of ouabain (red, ouabain), and upon washout (white, wash). Data from each experiment (open circles) and mean values ± SEM are shown. * and **—data differ significantly (*p* < 0.05 and *p* < 0.01, paired Student’s *t*-test).

In primary culture many neurons demonstrated the synchronization of miniature postsynaptic currents, leading to their summation into slow (up to several seconds) epileptiform currents (ECs) (Figure 1A). Generally those currents produce enough depolarization for neurons to reach the spike generation threshold. The frequency of ECs varies between cultures in the range of 0.05 to 3 events per second.

We first checked whether signaling effects occurring upon nanomolar ouabain action on NKA can modulate EC. In cells demonstrating EC, we performed whole-cell recording in control conditions and during the application of 1 nM ouabain (Figure 1B). Ouabain affected the shape of the ECs (Figure 1C), but the frequency of the ECs remained unchanged (Figure 1D and Table 1). The ouabain-induced decrease in the amplitude of the ECs (Figure 1E) expectedly reduced the integral inward current (Figure 1F and Table 1), since, at a constant EC frequency, the integral of the individual EC decreased (Figure 1G). Ouabain started to attenuate the EC amplitude almost instantly upon application and demonstrated a fully developed effect in 10–20 s (Figure 1B). EC amplitude remained reduced after ouabain washout (Figure 1G and Table 1). Therefore, NKA signaling can steadily attenuate but not block ECs.

**Figure 2:**
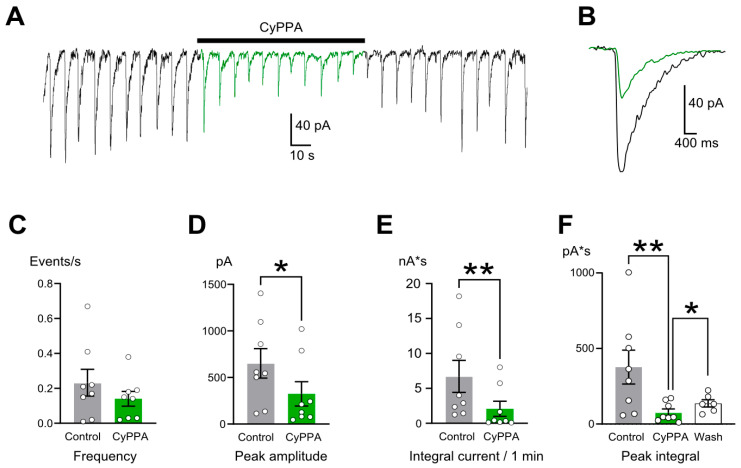
CyPPA (10 µM) reduces the magnitude of epileptiform currents (ECs). (**A**) A recording illustrating the developments of the effect of CyPPA (10 µM) on ECs. Application of CyPPA is shown as a bar above the trace. (**B**) An overlap of EPSCs averaged from intervals before (black) and during CyPPA application (green). (**C**–**F**) Comparison of mean ± SEM values of EC frequency (**C**), EC peak amplitudes (**D**), integral current per 1 min (**E**), and mean individual EC integral (**F**) before (gray, control), during application of CyPPA (green, CyPPA), and upon washout (white, wash). Data from each experiment (open circles) and mean values ± SEM are shown. * and **—data differ significantly (*p* < 0.05 and *p* < 0.01, paired Student’s *t*-test).

An activation of SK channels by 10 µM CyPPA (Figure 2A) also affected the shape of the ECs (Figure 2B). CyPPA did not affect the frequency of the ECs (Figure 2C and Table 1) but reduced their amplitude and integral current (Figure 2D–F). CyPPA’s effect on EC amplitude developed in 10–20 s. Unlike ouabain, CyPPA washout resulted in the partial restoration of the EC peak integral (Figure 2F and Table 1), demonstrating a reversible effect. Probably, washing out CyPPA cancels the hyperpolarizing effect of activation of SK channels.

### 2.2. NKA Signaling and SK-Channel Activation Inhibit Synchronized Calcium Transients in Neurons

**Figure 3:**
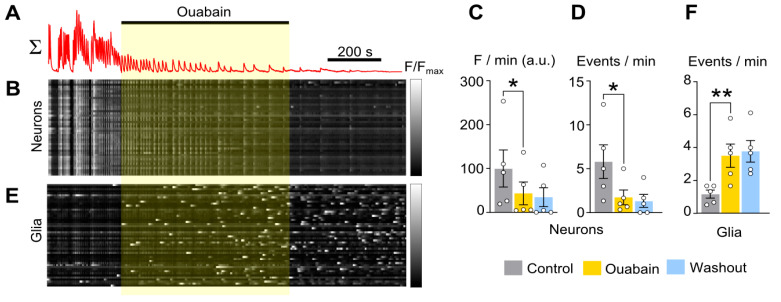
Ouabain (1 nM) reduces synchronized calcium oscillations of neurons. (**A**) Mean Fluo-8 intensity plotted over time for 50 neurons in 770 × 770 µM field of view shown above the (**B**) raster plot of normalized fluorescence intensity of individual neurons against time, grayscale coded according to fluorescence intensity. Rows correspond to individual neurons. Application of reagent is shown as a bar above the trace. (**C**,**D**) Average integral of intensity of neuronal fluorescence per 1 min (**C**) and frequency of neuronal calcium oscillations per 1 min (**D**) in control (gray), during ouabain application (yellow), and upon washout (blue). (**E**) Mean Fluo-8 intensity plotted over time for 50 glial cells. (**F**) Frequency of glial calcium oscillations per 1 min in control (gray), during ouabain application (yellow), and upon washout (blue). Data from each experiment (open circles) and mean values ± SEM are shown. * and **—data differ significantly (*p* < 0.05 and *p* < 0.01, paired Student’s *t*-test).

Optical registration of calcium events in a population of neurons revealed highly synchronized spontaneous calcium transients (CTs) involving the majority of neurons within the 770 × 770 µM field of view (FOV). For a whole FOV, the change in average fluorescence over time (Figure 3A) predominantly reflected the synchronized activity of neurons. A raster fluorescence intensity plot of individual neurons (Figure 3B) demonstrated high synchrony. Adding 1 nM ouabain reduced both the total fluorescence (Figure 3C and Table 2) of neurons and the frequency of CT generation (Figure 3D and Table 2). CT generation tended to vanish even after ouabain washout (Figure 3C,D and Table 2).

Because primary cortical neuron cultures also contain glial cells, we analyzed calcium events in glia as well. Unlike neurons, glia exhibited infrequent, long-lasting asynchronous calcium waves and were identifiable by their large, flat somas. The raster plot of activity of individual glial cells (Figure 3E) revealed rare oscillations in control. However adding ouabain triggered asynchronous spontaneous calcium transients in many glial cells (Figure 3F). An elevated frequency of glial calcium events was preserved upon ouabain washout (Figure 3E,F).

**Figure 4:**
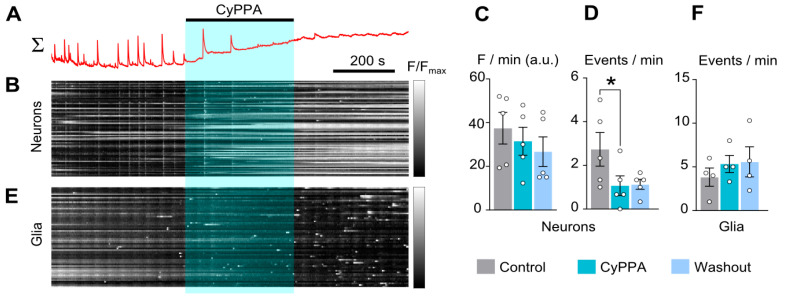
CyPPA (10 µM) reduces synchronized calcium oscillations of neurons. (**A**) Mean Fluo-8 intensity plotted over time for 50 neurons in 770 × 770 µM field of view shown above the (**B**) raster plot of normalized fluorescence intensity of individual neurons against time, grayscale coded according to fluorescence intensity. Rows correspond to individual neurons. Application of reagent is shown as a bar above the trace. (**C**,**D**) Average integral of intensity of neuronal fluorescence per 1 min (**C**) and frequency of neuronal calcium oscillations per 1 min (**D**) in control (gray), during CyPPA application (cyan), and upon washout (blue). (**E**) Mean Fluo-8 intensity plotted over time for 50 glial cells. (**F**) Frequency of glial calcium oscillations per 1 min in control (gray), during CyPPA application (cyan), and upon washout (blue). Data from each experiment (open circles) and mean values ± SEM are shown. *—data differ significantly (*p* < 0.05, paired Student’s *t*-test).

Adding 10 µM CyPPA on the top of spontaneous CT generation (Figure 4A,B) demonstrated an obvious decrease in CT amplitude but resulted in a slow elevation of intracellular calcium in many neurons (Figure 4B). In view of this circumstance, CyPPA did not decrease average calcium in the neuronal network (Figure 4C and Table 2) regardless of putting down the frequency of spontaneous CTs (Figure 4D). We did not observe any CyPPA effect of glial calcium transients (Figure 4E,F).

**Figure 5:**
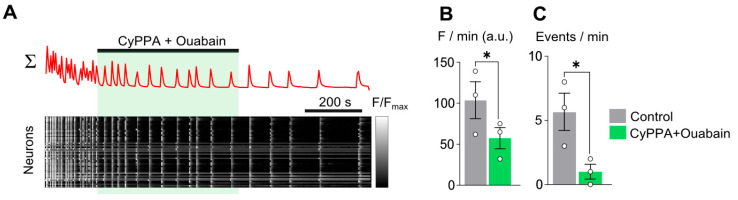
Combined application of CyPPA (10 µM) and ouabain (1 nM) reduces synchronized calcium oscillations of neurons. (**A**) Mean Fluo-8 intensity plotted over time for 50 neurons in 770 × 770 µM field of view shown above the raster plot of normalized fluorescence intensity of individual neurons against time, grayscale coded according to fluorescence intensity. Rows correspond to individual neurons. Application of reagents is shown as a bar above the trace. (**B**) Average integral of intensity of neuronal fluorescence per 1 min and (**C**) frequency of neuronal calcium oscillations per 1 min in control (gray) and during reagent application (green). Data from each experiment (open circles) and mean values ± SEM are shown. *—data differ significantly (*p* < 0.05, paired Student’s *t*-test).

The increase in background calcium with the action of CyPPA was unexpected. Given the ability of ouabain to reduce the amplitude of tonic calcium responses in neurons [14,15,16,28,29], we tested whether the CyPPA-induced elevation of intracellular calcium would occur when CyPPA is applied together with ouabain (Figure 5A). Co-application of ouabain with CyPPA reduced both the integral calcium-related fluorescence (Figure 5B and Table 2) and the frequency of CTs (Figure 5C and Table 2).

**Figure 6:**
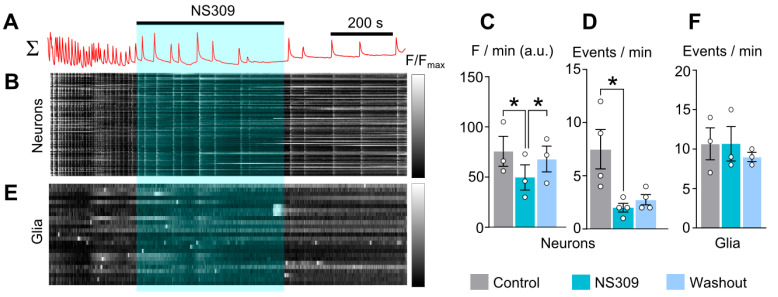
NS309 (10 µM) reduces synchronized calcium oscillations of neurons. (**A**) Mean Fluo-8 intensity plotted over time for 50 neurons in 770 × 770 µM field of view shown above the (**B**) raster plot of normalized fluorescence intensity of individual neurons against time, grayscale coded according to fluorescence intensity. Rows correspond to individual neurons. (**C**,**D**) Average integral of intensity of neuronal fluorescence per 1 min (**C**) and frequency of neuronal calcium oscillations per 1 min (**D**) in control (gray), during NS309 application (cyan), and upon washout (blue). (**E**) Mean Fluo-8 intensity plotted over time for 50 glial cells. (**F**) Frequency of glial calcium oscillations per 1 min in control (gray), during NS309 application (cyan), and upon washout (blue). Data from each experiment (open circles) and mean values ± SEM are shown. *—data differ significantly (*p* < 0.05 paired Student’s *t*-test).

For comparison, we tested whether NS309, another activator of SK channels, will also affect baseline calcium and CT frequency. Adding 10 µM NS309 (Figure 6A,B) decreased the average calcium fluorescence (Figure 6C and Table 2) as well as the frequency of CTs (Figure 6D and Table 2). Unlike CyPPA, NS309 did not cause tonic elevation of intracellular calcium, which may be due to activation of IK channels in addition to SK channels. NS309 also did not affect calcium transients in glial cells (Figure 6E,F).

## 3. Discussion

This study provides compelling evidence that low-nanomolar concentrations of ouabain, through the selective modulation of NKA, can attenuate epileptiform neuronal activity by engaging intracellular signaling mechanisms that restrict calcium dynamics. Particularly, we show that 1 nM ouabain significantly reduces the amplitude and integral of ECs and suppresses synchronized CTs in cortical neuronal cultures. These results suggest that NKA functions not only as an electrogenic pump but also as a signaling hub that modulates neuronal excitability via downstream pathways.

The ECs recorded under magnesium-free conditions reflect synchronized bursts of excitatory postsynaptic currents, likely driven by recurrent synaptic vesicle release and NMDA receptor activation [30,31,32,33]. This form of self-organized network activity is associated with calcium influx, both through voltage-gated calcium channels and synaptic receptors. We found that the ouabain-induced modulation of NKA activity reduces the amplitude of ECs without altering their frequency, indicating that, while the initiation of synaptic release remains intact, the resulting depolarization and associated calcium entry are diminished.

Calcium imaging demonstrated that ouabain diminished EC amplitude and profoundly suppressed both the occurrence and intensity of CTs in neuronal populations. This supports the hypothesis that low-dose ouabain initiates intracellular signaling cascades that decouple synaptic activity from widespread calcium elevation. Since CTs are essential for driving spontaneous synaptic activity and maintaining network synchrony, their reduction likely leads to diminished excitatory feedback and reduced epileptiform propagation. These effects were long lasting and persisted after ouabain washout, further suggesting that NKA signaling induces a durable shift in the excitability state of the neuronal network.

The nature of this intracellular signaling remains to be fully elucidated, but at low (subnanomolar–nanomolar) concentrations, ouabain facilitates functional NKA–NCX ionotropic coupling within membrane microdomains [13,29], allowing NCX to operate in the forward mode and accelerate Ca^2+^ extrusion from the cytosol [14,16]. Pharmacological blockade of NCX (e.g., with KB-R7943) abolishes this effect, confirming its role in ouabain-mediated Ca^2+^ lowering [14]. This mechanism likely underlies the ability of low ouabain concentrations to reduce NMDA- or kainate-induced Ca^2+^ transients in neurons, thereby preventing excitotoxic Ca^2+^ overload [14,15,16,17]. In parallel, ouabain functions as a signaling ligand for NKA, activating Src, MAPK, PKA, and PKC pathways, which further contribute to decreased Ca^2+^ influx and/or enhanced extrusion [14,16,21,34,35].

Notably, the glial response to ouabain—characterized by asynchronous calcium transients—suggests a broader effect of NKA signaling on neuron–glia communication, which may contribute to overall network stabilization. As 1 nM ouabain may facilitate activity of glial NKA isoforms [13], it can improve the ion gradients that drive glutamate uptake via EAAT1/EAAT2 and K^+^ clearance through Kir4.1. Because Kir4.1 dysfunction promotes seizures [36,37,38,39], NKA facilitation can help prevent them by stabilizing extracellular K^+^ and limiting hyperexcitability. Thus, ouabain’s modulation of glial homeostasis may indirectly influence neuronal activity, while increased astrocytic Ca^2+^ spiking is not directly linked to ion gradient maintenance but may serve as a marker of ouabain’s action on glia.

In contrast to ouabain, CyPPA, an SK2/3-selective activator, reduced the amplitude of ECs and suppressed CT frequency without decreasing overall calcium levels. While CyPPA enhanced medium afterhyperpolarization (mAHP) and limited synchronized bursting, it unexpectedly induced a gradual elevation in baseline intracellular calcium. This effect has not been previously observed by other investigators and may be due to the off-target effect of CyPPA on mitochondrial Ca^2+^ uptake [12]. Interestingly, co-application of ouabain abolished this calcium elevation, reinforcing the idea that NKA signaling not only suppresses synaptic transmission but also regulates intracellular calcium buffering [14,29].

Our findings were further validated using NS309, a broader SK/IK channel modulator, which reduced both CT frequency and basal calcium levels without affecting glial cells. Since CyPPA and NS309 share a similar mode of action, but only CyPPA increased baseline calcium levels, the effect on basal Ca^2+^ we observed is likely a CyPPA-specific side effect unrelated to SK-channel activation.

Generally our findings highlight the therapeutic potential of SK-channel activation in suppressing network hyperexcitability and the potential for synergistic effects of drugs acting on SK channels and NKA.

Taken together, the data suggest a model in which low concentrations of ouabain reduce intracellular calcium elevation by inhibiting calcium influx associated with synchronized ECs. This may be achieved through modulation of the Na/Ca exchanger [14,32] and intracellular signaling pathways downstream of NKA [14,16,34,35], leading to reduced synaptic vesicle release [29] probability or dampened excitatory postsynaptic potential [40]. By limiting CTs, ouabain appears to diminish spontaneous excitatory drive within the network—a desirable feature for antiepileptiform interventions.

This study was conducted using an in vitro model of neuronal hyperexcitability. Further in vivo studies are needed to confirm the relevance of NKA-mediated signaling pathways. Future investigations should also identify the specific downstream effectors of NKA activation by nanomolar ouabain using selective pharmacological blockers. It should be noted that primary neuronal cultures cannot fully capture complex network phenomena or interactions between different brain regions. While this approach is valuable for studying cellular and synaptic mechanisms, it has limited relevance for network-level activity, chronic disease progression, and systemic influences. Therefore, it is best used in combination with animal models. Identifying cellular mechanisms that regulate synchronized neuronal network activity—shared across different network types—could help develop therapies targeting common network processes and offer new options for treating drug-resistant epilepsy.

Although the pharmacological agents used in this study serve primarily as experimental tools rather than as therapeutic drugs, our findings reveal a novel mechanism by which low concentrations of ouabain suppress epileptiform activity. This effect is mediated through NKA-dependent signaling pathways that reduce EC-induced calcium influx and attenuate network-wide calcium synchronization. These findings suggest that NKA is a promising target for antiepileptiform therapies, particularly when combined with modulators of SK channels that act through complementary hyperpolarization mechanisms. The convergence of these distinct pathways offers a rational basis for the development of combination strategies to combat drug-resistant epilepsy.

## 4. Materials and Methods

### 4.1. Primary Culture of Cortical Neurons

All manipulations on animals were performed in accordance with the guide of the Federation for Laboratory Animal Science Associations and were approved by the Animal Care and Use Committees of Sechenov Institute (Protocol 1–6/2022 from 21 January 2022). Briefly, 16 days pregnant Wistar rats (14 female rats used in this study, supplied by the Sechenov Institute Animal Facility) were placed in a plastic box connected to a CO_2_ tank by a tube and then sacrificed by 1 min CO_2_ inhalation. Fetuses were removed, and then primary cultures of rat cortical neurons were prepared using conventional procedures as described earlier [41]. Neurons were grown in Neurobasal culture medium supplemented with B-27 (Paneco Ltd., Moscow, Russia) on glass 15 mm diameter coverslips coated with poly-D-lysine and were used for experiments after 12–14 days in culture.

### 4.2. Recording of Postsynaptic Currents and Data Processing

Whole-cell currents were recorded from cultured rat cortical neurons using a MultiClamp 700B patch-clamp amplifier (Molecular Devices, San Jose, CA, USA). Signals were low-pass filtered at 400 Hz and digitized at a sampling rate of 20 kHz using a Digidata 1440A interface, controlled by pClamp v10.6 software (Molecular Devices, San Jose, CA, USA). Solution exchange was performed using a fast application system, as previously described [42].

Unless stated otherwise, the external bathing solution contained (in mM): 144 NaCl, 2.8 KCl, 1.0 CaCl2, and 10 HEPES, adjusted to pH 7.2–7.4 with an osmolarity of 310 mOsm. Magnesium was removed from the perfusion solution to prevent the potential-dependent block of NMDA receptors [43] and provoke the generation of synchronized neuronal activity. The intrapipette (internal) solution contained (in mM): 120 CsF, 10 CsCl, 10 EGTA, and 10 HEPES, adjusted to pH 7.4 with CsOH and osmolarity of 300 mOsm. Patch pipettes with a resistance of 4–6 MΩ were pulled from RWD B-15086-10F borosilicate glass capillaries with filament.

All experiments were conducted at 24–26 °C. To record mEPSCs, neurons were voltage-clamped at –70 mV.

Epileptiform events were automatically detected in ClampFit v10.6 (Molecular Devices). Before event detection we performed drift correction to eliminate slow baseline shifts at a timescale of minutes. The frequency of events, mean amplitude, time constants for rise and decay, integral current were measured for control conditions, in the presence of the test substance, and after washout. One recording was considered one experiment in statistics.

### 4.3. Optical Detection of Calcium Transients and Image Processing

Cortical neurons grown on 15 mm diameter glass coverslips were loaded with the Ca^2+^-sensitive dye Fluo-8 by incubation in the basic solution (in mM: 144 NaCl, 2.8 KCl, 2 CaCl_2_, 10 HEPES; pH 7.4; osmolarity 310 mOsm) containing 2 µM Fluo-8 acetoxy-methyl ester (Fluo-8 AM, Abcam, Cambridge, UK) at room temperature for 60 min, followed by a 20 min washout with the basic solution. Coverslips were then placed onto the stage of a Leica SP5 MP inverted microscope (Leica Microsystems, GmbH, Wetzlar, Germany) and continuously perfused with the basic solution at a flow rate of 1.2 mL/min.

Reagents were applied using a fast perfusion system enabling rapid solution exchange. Fluorescence was excited with a 488-nanometer laser and detected within the 510–560 nm range at approximately 660 ms intervals (1024 × 1024 pixel frame, 1.5 fps, 20× oil immersion objective, 770 × 770 µM field of view). Image time series were captured using Leica LAS AF v2.3.6 software (Leica Microsystems GmbH, Wetzlar, Germany). All experiments were conducted at 24–26 °C.

For analysis, image time series were exported to NetCal 8.4.1 software (https://github.com/orlandi/netcal, accessed on 1 October 2025) [44]. We performed “Standard preprocessing” and unsupervised “Automatic ROI detection” by threshold. NETCAL extracted fluorescence traces (fluorescence intensity changes over time) from approximately 100 to 400 cells within one field of view. Traces of individual ROIs were manually classified into 3 groups: (1) neurons—periodic events with sharp rise and slow decay; (2) glia—rare asynchronous events and slow wave events of cells with large, flat bodies; (3) noise—low amplitude, Ca^2+^ overloaded, and other artifacts. For neurons and glia, the event frequency and integral Ca^2+^ entry were measured for control, in the presence of the test-substance, and after washout. One recording captured from one coverslip was considered one experiment in statistics.

### 4.4. Reagents

In both patch-clamp and imaging experiments CyPPA 10 µM (*N*-cyclohexyl-*N*-[2-(3,5-dimethyl-pyrazol-1-yl)-6-methyl-4-pyrimidinamine)—a K_Ca_2.2 and K_Ca_2.3 (SK) channel activator—or 10 µM NS309 positive modulator of small- (SK) and intermediate- (IK) conductance Ca^2+^-activated K^+^ channels (K_Ca_2 and K_Ca_3.1 channels), or 1 nM ouabain were applied using a local perfusion system, achieving full solution exchange in 10 s. Reagents were obtained from (Abcam, Cambridge, UK).

### 4.5. Statistics

The data are presented as representative measurements as well as mean values ± standard error of the mean (SEM). For patch-clamp recordings the sample number (*n*) refers to the number of recorded cells (each from a separate coverslip). For imaging experiments the sample number (*n*) refers to the number of captured coverslips. Data pairs were compared using Student’s two-tailed *t*-test. Statistical significance is reported in the figures according to the following symbols: * and **, which indicate *p* values below (<) 0.05 and 0.01, respectively.

## Figures and Tables

**Table 1:** Average values for individual EC integral current (pA × s).

	Control	Application	Washout
Ouabain	333 ± 102 (*n* = 11)	137 ± 25 (*n* = 11) *	132 ± 40 (*n* = 5)
CyPPA	376 ± 112 (*n* = 8)	77 ± 22 (*n* = 8) **	136 ± 23 (*n* = 6) #

*, **—data are significantly different from control (*p* < 0.01 and *p* < 0.05); #—data are significantly different from application (*p* < 0.05).

**Table 2:** Quantitative values for optically detected parameters of frequency and mean intensity (a.u.) of neuronal synchronized calcium transients caused by ECs.

	Intensity/Min			Events/Min		
	Control	Application	Washout	Control	Application	Washout
Ouabain 1 nM	100 ± 42 (*n* = 5)	43 ± 26 (*n* = 5) *	34 ± 21 (*n* = 5)	5.8 ± 1.9 (*n* = 5)	1.7 ± 0.8 (*n* = 5) *	1.3 ± 0.7 (*n* = 5)
CyPPA 10 µM	37 ± 7.2 (*n* = 5)	31 ± 6.4 (*n* = 5)	27 ± 6.7 (*n* = 5)	2.7 ± 0.8 (*n* = 5)	1.0 ± 0.45 (*n* = 5) *	1.1 ± 0.2 (*n* = 5)
CyPPA 10 µM + Ouabain 1 nM	103 ± 22 (*n* = 3)	57 ± 13 (*n* = 3) *		5.6 ± 1.4 (*n* = 3)	1.0 ± 0.5 (*n* = 3) *	
NS309 10 µM	76 ± 15 (*n* = 3)	50 ± 13 (*n* = 3) *	68 ± 13 (*n* = 3) #	8.3 ± 2.3 (*n* = 4)	2.0 ± 0.5 (*n* = 4) *	3.0 ± 0.6 (*n* = 4)

*—data are significantly different from control; #—data are significantly different from application.

## Data Availability

The original contributions presented in the study are included in the article; further inquiries can be directed to the corresponding author.
